# Examining the Association Between Episiotomy and Severe Perineal Tears in a Tertiary Care Center Implementing a Restrictive Episiotomy Policy

**DOI:** 10.7759/cureus.31606

**Published:** 2022-11-17

**Authors:** Yaser A Faden, Azizah M Fatani, Batoul M Fallatah, Tala S Rawa, Shatha A Almasri, Nadia O El Amin, Sondos A Rawas, Mohammed Y Al-Hindi

**Affiliations:** 1 Obstetrics and Gynecology, King Abdulaziz Medical City-WR, Ministry of National Guard Health Affairs, Jeddah, SAU; 2 College of Medicine, King Saud Bin Abdulaziz University for Health Sciences, Jeddah, SAU; 3 Research Committee, King Abdullah International Medical Research Center, Jeddah, SAU; 4 Pediatrics, King Abdulaziz Medical City-WR, Ministry of National Guard Health Affairs, Jeddah, SAU

**Keywords:** perineal tear, parity, restrictive episiotomy, 3rd degree tear, severe laceration, episiotomy

## Abstract

Background

Perineal lacerations are feared complications of vaginal delivery, especially the severe types (third- and fourth-degree tears). World Health Organization (WHO) recommended restrictive episiotomy practice after alarming literature linked the increase in severe tears with routine episiotomy. Therefore, this study aimed to measure the association between episiotomy and the incidence of third- and fourth-degree perineal tears and infections in women who underwent episiotomy versus those who did not at a tertiary care center implementing the restrictive episiotomy policy in Jeddah, Saudi Arabia.

Methods

This retrospective cohort study was conducted in the Department of Obstetrics and Gynecology at King Abdulaziz Medical City (KAMC), Western Region, between May 2016 and May 2018, targeting all pregnant women who underwent normal spontaneous vaginal delivery. The nonprobability convenient sampling technique was used for women who underwent episiotomy. Women without episiotomy (control group) were randomly selected in a 1:1 ratio. The prevalence (incidence) of episiotomy and its association with severe perineal tears were measured. Statistical data were analyzed using SPSS version 27 (IBM Corp., Armonk, NY). A *p*-value of less than 0.05 was considered significant.

Result

A total of 7436 deliveries were recorded. At KAMC, episiotomy had a prevalence of 10% and was more common in primipara. The incidence of third-degree tears was 3.3% in the episiotomy group and 0.8% in the control group (odds ratio, 4.1; *p* = 0.03). None had fourth-degree tears. Furthermore, the infection rate was not significantly different between the two groups (0.1% vs. 0.1%). Using Firth's logistic regression model, primipara emerged as an independent significant risk factor (OR, 3.5 [1.1-11.2]; *p* = 0.035) while the trend toward increased risk for tear development in the episiotomy group became statistically insignificant (OR, 2.3 [0.7-8.0]; *p *= 0.19). A post hoc examination to observe the association between episiotomy exposure and BMI using a stepwise logistic regression model showed that parity and age were independent risk factors for episiotomy, with OR values of 2.2 (1.6-3.2) and 0.9 (0.88-0.94), respectively (*p* < 0.001). The BMI became insignificant, with an OR of 1.0 (0.7-1.4) (*p *= 0.96).

Conclusion

The development of severe perineal tears in a center with a restrictive episiotomy policy is rare. Parity has emerged as an independent risk factor for severe perineal tears. Prospective multicenter research with a larger sample size is recommended to validate this study's findings further and investigate other obstetric measures to reduce severe tears in primi mothers.

## Introduction

Perineal lacerations are suspected complications of vaginal delivery. These lacerations, or tears, are divided into four degrees, depending on the extensiveness of their depth into the perineal tissues. The first-degree tear is the least severe; it occurs in many deliveries and involves only the lining of the vagina. If the tear reaches the perineal muscles, it is a second-degree tear. The first and second-degree tears are considered minor tears. In undesirable conditions, third- and fourth-degree tears might occur. Third-degree tears extend to the anal sphincter. A further extension to the rectal mucosa results in a fourth-degree tear.

Episiotomy was thought to be a preventive measure [1-3]. However, the WHO emphasizes that the routine/liberal use of episiotomy is “not recommended”; they only recommend selective/restrictive use [4]. A systematic review reported that the prevalence of episiotomy practice in the Middle East is 67% [5]. In Saudi Arabia, routine episiotomy is highly prevalent, ranging from 36% to 51% [6,7]. In other parts of the world, episiotomy is becoming a restrictive practice [8].

In a systematic review of observational studies, episiotomy was associated significantly with women with severe perineal tears. Studies that examined the association of episiotomy with third- and fourth-degree tears in the region were retrospective case-control studies, and they were only a few [9,10]. Our center is one of the few tertiary care maternity units implementing the restrictive episiotomy policy even before the WHO recommendations.

Thus, this study aimed to measure the prevalence (incidence) of episiotomy and its association with third- and fourth-degree perineal tears among women admitted to a tertiary care center implementing a restrictive episiotomy policy in Saudi Arabia.

## Materials and methods

This retrospective cohort study included women who underwent normal spontaneous vaginal delivery (NSVD) at the Department of Obstetrics and Gynecology in a tertiary care center, King Abdulaziz Medical City (KAMC), in Jeddah, Saudi Arabia, between May 2016 and May 2018. This institution conducts approximately 3500 deliveries annually. The Institutional Review Board Office of the King Abdullah International Medical Research Center approved this study with reference number IRBC/0938/19.

The inclusion criteria were as follows: NSVD, age between 18 and 45 years, any parity, and gestational age of 37 weeks or more. Conversely, the exclusion criteria were the following: preterm delivery, instrumental delivery, fetuses with macrocephaly, and shoulder dystocia. Women with chronic or gestational-related co-morbidities were not excluded. The eligible women were compared with women who underwent NSVD without episiotomy (control group).

The sample size was calculated according to a previous systematic review that showed that severe tears were 30%-40% less in the non-episiotomy group than in the episiotomy group [11]. Hence, 354 participants (cases) in each group were needed to detect a statistically significant difference based on a confidence level of alpha 95 and power of (1-beta) of 80% and a population of 4478 [12]. For the sampling technique, the case group was selected via a nonprobability convenient sampling technique, whereas the control group was randomly selected with a 1:1 ratio and matched with the cases according to age and parity.

The data were collected from the electronic hospital information system (BestCare system) and validated with the labor and delivery unit's case records (log book). The study variables were collected into data sheets, including maternal demographics and perinatal events.

The data were screened for errors and missing variables and entered into SPSS® version 27 for analysis. Data are expressed using proportions for categorical and mean, median, standard deviation, and interquartile range (IQR) depending on the normality of the distribution. For the univariate analysis of the demographic characteristics and primary outcomes, the associations were calculated using chi-square or Fisher’s exact tests accordingly. For the continuous variables, the student’s t-test and Mann-Whitney U test were used, depending on the normality of the distribution. Binary outcomes with small samples were analyzed using Firth’s logistic regression to assess for significant predictors. While it reduces bias in the maximum likelihood estimates of coefficients, bias toward one-half is introduced in the predicted probabilities [13]. We conducted a post hoc examination using a stepwise logistic regression model to examine the association between episiotomy exposure and BMI. A p-value of less than 0.05 was considered significant.

## Results

**Figure 1:**
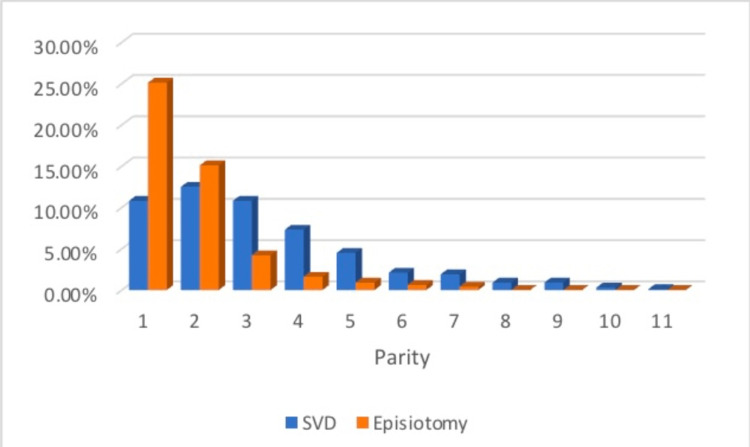
The distribution rates of parity and episiotomy in the included sample SVD: spontaneous vaginal delivery

During the study period, 7436 deliveries were recorded, and 4478 (60.2%) of them were NSVDs. A total of 452 episiotomies were performed (prevalence: 10%). We excluded 11 preterm deliveries and 79 cases that had missing essential data. Hence, 362 episiotomy cases were ultimately included. Meanwhile, we randomly selected 362 women in the control group for a 1:1 ratio. Table 1 shows the maternal and neonatal characteristics in both groups. In comparing the two groups, the episiotomy group had a significantly lower age (mean (SD) 25.8 (5.0) years vs. 29 (5.7) years) and parity than the non-episiotomy group (median (IQR) 0 (0-1) vs. 2 (1-3)) (p < 0.001). Episiotomy was performed more commonly in primipara (Figure 1).

**Table 1:** Maternal and neonatal characteristics * Mean (standard deviation), ** n (%), ***Median (interquartile range). NSVD, normal spontaneous vaginal delivery; BMI, body mass index; NBW, normal birth weight; LBW, low birth weight; LGA, large for gestational age

Clinical Characteristics	SVD (N = 362)	Episiotomy (N = 362)	p-value
Maternal age, years*	29.2 (5.7)	25.8 (5.0)	<0.001
BMI*	28.6 (5.6)	27.6 (5.4)	0.02
BMI < 30**	209 (60.4)	237 (68.7)	0.03
BMI ≥ 30	137 (39.6)	108 (31.3)	
Parity***	2 (1–3)	0 (0–1)	<0.001
Primipara	73 (20.8)	169 (52.3)	<0.001
Multipara	278 (79.2)	154 (47.7)	<0.001
Age of gestation (weeks)*	39.1 (1.1)	39.1 (1.1)	0.24
Birth weight (kg)*	3.1 (0.4)	3.0 (0.4)	0.24
NBW	321 (92)	310 (88.8)	0.24
LBW	20 (5.7)	32 (9.2)	0.24
LGA	8 (2.3)	7 (2)	0.24

 

In addition, the body mass index (BMI) was significantly higher in the control group than in the episiotomy group (39.6% with BMI ≥ 30: 39.6% vs. 31.3%, p = 0.002). Regarding the neonatal characteristics, the gestational age at birth was comparable between the two groups as well as the birth weight subcategories, as shown in Table 1.

Tears were reported in 323 (44.6%) women, of which the first, second, and third degrees were 184 (25.4%), 124 (17.1%), and 15 (2.1%), respectively. The groups were recategorized to represent women with no or minor tears 709 (97.9%) and those with severe tears 15 (2.1%). None had experienced fourth-degree tears.

For the primary outcome, the unadjusted association analysis showed that the episiotomy group was more likely to have severe tears than the control group (odds ratio (OR), 4.2; 95% confidence interval (CI) = 1.2-14.7; p = 0.03).

Using Firth’s logistic regression model, we included the significant variables in the univariate analysis. The model included the severity of the tear as a dependent variable and episiotomy, parity, and BMI as independent risk factors. Maternal age was included in the model because it highly correlated with parity, with a polychoric correlation coefficient (r) of 0.7. Being primipara was also an independent significant risk factor (OR, 3.5 (1.1-11.2); p = 0.035). However, the trend toward an increased risk for tear development in the episiotomy group became statistically insignificant (OR, 2.3 (0.7-8.0); p = 0.19). The BMI was not associated with severe tears (OR, 0.8 (0.2-2.6), p = 0.65). Furthermore, the model was found to be statistically significant (penalized likelihood = −60.2, Wald = 8.8; p = 0.03).

We conducted a post hoc examination to observe the association between episiotomy exposure and BMI. We used a stepwise logistic regression model where the independent variable was episiotomy, and the independent variables were age, BMI, and parity. Parity and age were independent risk factors for episiotomy, with OR values of 2.2 (1.6-3.2) and 0.9 (0.88-0.94), respectively (p < 0.001). The BMI became insignificant, with an OR of 1.0 (0.7-1.4) (p = 0.96).

Regarding the secondary outcome, the prevalence of having perineal infections in the episiotomy group was 4 (1.1%), whereas that in the control group was 3 (0.8%) (p = 1.00). The overall infection rate was 1% (7/724). None of the women had perineal bleeding.

## Discussion

This study measured the association between episiotomy and severe tears in a tertiary care center implementing restrictive episiotomy practice. The episiotomy prevalence among women who underwent NSVD was 10%, which is lower than that of other Saudi Arabia and Middle Eastern centers. In Oman and Turkey, the episiotomy rate was 66% and 56.3%, respectively [14,15]. A systematic review of 12 observational studies estimated that 57% of 69,171 women from the Middle East underwent episiotomy [5]. In Saudi Arabia, the episiotomy rate ranged from 36.4% to 51.20% [6,7].

In 2013, the American College of Obstetricians and Gynecologists recommended that the use of episiotomy should be restricted in clinical practice because of the high rates of injury [16]. In addition, the WHO emphasizes that the routine/liberal use of episiotomy is "not recommended" while recommending its selective/restrictive use [4]. In many developing countries, episiotomy has been a routine practice in all women in labor to avoid severe perineal tears, as stated above. However, many countries, such as France, adopted the French Guidelines' restrictive policy on episiotomy in 2005. Consequently, the episiotomy rates dramatically declined from 55.7% in 2004 to 13.3% in 2009. Some centers even achieved a rate of 7.6% by implementing restrictive practices [8]. The prevalence of episiotomy of 10% in our center is not far from the international figures for restrictive use [17]. Since 2000, we have implemented restrictive episiotomy practices, and to our knowledge, this center is the first to report such prevalence in Saudi Arabia and the Middle East.

As noticed in the maternal characteristics, women exposed to episiotomy tended to be younger, similar to that reported in another study in Saudi Arabia [10] and not different from various international figures [8]. Alongside age is parity. Most episiotomies occurred in primipara. Such practice is done out of fear of perineal tears and to facilitate delivery, as believed by some physicians based on the first episiotomy in earlier-century studies. The practice of episiotomy has been expanded and routinely done in some centers after a published paper in 1921 claimed that episiotomy could save the fetal brain from injury, epilepsy, and cerebral palsy [1-3]. Also documented in the literature, severe tears occur almost four times more commonly in primipara [11]. Hence, episiotomy practice in primipara is augmented with such fear, and weaning it off is difficult in many parts of the world.

Moreover, maternal BMI and its association with episiotomy are rarely discussed in the literature. The association seems to follow a linear paradigm; given that the primipara mothers are younger, they consequently have less BMI. However, such a concept has not been challenged. In this study, women with higher BMI were less exposed to episiotomy; in other words, women with lower BMI were more exposed to episiotomy. Hence, we conducted a post hoc examination to observe the association between episiotomy exposure and BMI. We used a stepwise logistic regression model; as expected, lower parity and age were independent risk factors for episiotomy. The BMI was not associated with episiotomy exposure. We found no other studies, to our knowledge, that have examined such an association.

In this study, neither gestational age nor birth weight was associated with episiotomy exposure, probably because of the study restriction, with the exclusion of deliveries complicated by assisted vaginal delivery, shoulder dystocia, or macrocephaly.

In contrast, we found an association between episiotomy and the occurrence of severe perineal tears in the unadjusted analysis. However, once risk factors were considered using regression analysis, the association became statically insignificant but remained to show an increasing trend [11]. This result could be explained by the low prevalence of routine episiotomy in our center because of the restrictive policy. Therefore, it will be underpowered to detect a statistically significant association. Further, parity became an independent risk factor that increased the odds of severe tears. A systematic review included eight trials with 5404 women who underwent restrictive episiotomy compared with routine episiotomy. The restrictive use of episiotomy showed a lower risk of clinically relevant morbidities, including severe perineal trauma (relative risk (RR), 0.67 (0.49-0.91)) and posterior perineal trauma (RR, 0.88; (0.84-0.92)). The only disadvantage shown in the restrictive use of episiotomy was an increased risk for anterior perineal trauma (RR, 1.84 (1.61-2.10)) [17]. Such a finding in this meta-analysis explains the decreased prevalence of perineal trauma in our center (2%), considering that we are applying the restrictive policy. Thus, offsetting the initial association with a wide 95% CI, once parity was included in the regression model, it manifested as an independent risk factor. Finally, perineal infections were rare, consistent with the literature [18].

To our knowledge, this study is the first to report the prevalence of episiotomy and examine its association with severe tears in a tertiary care center that has applied a restrictive episiotomy policy in Saudi Arabia. Additionally, it is the first to investigate the association between maternal BMI and the performance of episiotomy. Hence, it could be used as a benchmark for further quality improvement studies in other national centers.

However, this study has some limitations. First, considering the study's nature, selection bias due to missing data could affect the results. Moreover, the low outcome prevalence might dilute the association; hence, more extensive, multicenter prospective cohort studies are warranted. Research on the practice of episiotomy in Saudi Arabia and its relation to maternal antenatal and socioeconomic status using the multidomain scoring system is recommended [19,20]. Future studies should investigate other obstetric measures to anticipate and prevent severe perineal tears directed by international guidelines [1]. Such a future outlook should provide a holistic one-stop research article on restrictive episiotomy approaches. Finally, the effect of such a policy should have a long-term follow-up to determine the sequelae on maternal, infant, and healthcare resource utilization [19-22].

## Conclusions

Development of severe perineal tears and infections in a center with a restrictive episiotomy policy is rare. Parity has emerged as an independent risk factor for severe perineal tears. Prospective multicenter research with a larger sample size is recommended to further validate this study's findings and investigate other obstetric measures to reduce severe tears in primi mothers.
